# Unanticipated Difficult Airway Consistent With Suspected Subglottic Obstruction in an Asthmatic Patient

**DOI:** 10.7759/cureus.113276

**Published:** 2026-07-24

**Authors:** Afnan Amjad, Nader Al-Mane, Anas El-Burki

**Affiliations:** 1 Department of Anesthesia and Critical Care, Naas General Hospital, Naas, IRL

**Keywords:** bougie, difficult airway, subglottic tracheal narrowing, sugammadex, video-assisted laryngoscopy

## Abstract

Unexpected difficult airway during elective surgery under general anesthesia is one of the most critical problems in perioperative medicine. We report the case of a 68-year-old female patient of Somali origin (body mass index 31.2 kg/m^2^), with type 2 diabetes mellitus controlled with insulin, hypercholesterolemia, and mild persistent asthma, who was scheduled for elective incisional hernia repair. The patient underwent preoperative airway assessment involving Mallampati Class II; clinically normal mouth opening and unrestricted neck mobility did not predict difficulty. Previously, a history of severe, prolonged cough and sore throat that lasted for around 12 hours after she underwent cholecystectomy in Somalia five years ago was reported but not interpreted as an airway predictor. After administering midazolam 2 mg, fentanyl 150 ug, propofol 200 mg, and rocuronium 70 mg, video laryngoscopy was used to find the vocal cords, but it was unsuccessful. Respectively, attempts to intubate using two bougie-assisted 7.5 and 7.0 mm endotracheal tubes were unsuccessful twice because of subglottic resistance. Ultimately, the senior anesthetist succeeded in intubation using a size 7.0 mm endotracheal tube with a controlled clockwise-to-anticlockwise rotational tube technique, consistent with the underlying subglottic narrowing of the trachea. Peripheral capillary oxygen saturation (SpO₂) was maintained at 97% and above, and total time to confirmed intubation from start of induction was around nine minutes. Sugammadex 200 mg was administered at the end of surgery; quantitative neuromuscular monitoring was not performed, and the patient was extubated fully awake, meeting clinical extubation criteria (fully awake, obeying commands, SpO₂ >97% on spontaneous ventilation) without the development of any airway-related complications.

## Introduction

During elective surgeries, an unanticipated difficult airway is one of the most significant challenges and a potentially catastrophic event in anesthetic practice. In spite of systematic preoperative assessment using standardized tools such as the Mallampati classification, thyromental distance, interincisor gap, neck mobility, and the Look externally, Evaluate the 3-3-2 rule, Mallampati classification, Obstruction, Neck mobility mnemonic, a significant proportion of airway complications remain unidentified without performing laryngoscopy [1]. The Difficult Airway Society (DAS) 2025 guidelines recommend a linear algorithm progressing through plans (A-D), with focus on continuous oxygenation and early escalation as cardinal principles [2]. Moreover, the 2022 American Society of Anesthesiologists Practice Guidelines also stress team-based responses and proactive planning [3]. However, anatomical differences, which are undetectable on external examination, particularly the subglottic tracheal narrowing, may result in transforming a predicted straightforward intubation into a life-threatening emergency.

Subglottic stenosis, postintubation, inflammatory or idiopathic, is a well-known cause of unexpected intubation difficulty. In their case report, Hibino et al. described asymptomatic subglottic stenosis discovered during anesthetic induction in a 74-year-old woman scheduled for elective orthopedic surgery in whom subglottic resistance was encountered despite entirely normal preoperative assessment [4]. Importantly, unrecognized subglottic stenosis may manifest only with asthma-like symptoms or with unexplained dyspnea, and the cross-sectional area of the trachea must be reduced by over 50% before audible stridor becomes apparent [5]. In asthmatic patients, such symptoms are directly attributed to bronchospasm rather than mechanical obstruction, further increasing the chances of missed diagnosis.

The patient's past anesthetic history is a resource that is often overlooked for obtaining preoperative airway information. Postoperative sore throat is considered to be a minor, self-limiting postoperative complication, occurring in as many as 32.4% of patients after endotracheal intubation [6]. Nevertheless, if postoperative sore throat is severe or occurs for an extended duration (beyond the usual two to six hours after extubation), it could be a sign of previous subglottic problems. This case illustrates that careful attention to previous anesthetic symptoms may provide the single most actionable preoperative predictor of difficult airway in patients with no other apparent risk factors.

## Case presentation

A 68-year-old female patient with a body mass index of 31.2 kg/m² (height 160 cm; weight 80 kg) was planned for an elective incisional hernia repair under general anesthesia. Her underlying medical history included controlled type 2 diabetes mellitus, hypercholesterolemia (treated with atorvastatin), and mild persistent asthma (treated with salbutamol as needed). Additionally, she was a nonsmoker with no known drug allergies. Airway assessment was carried out preoperatively, which showed Mallampati Class II, clinically normal mouth opening, and unrestricted neck mobility; none of the standard criteria for predicted difficult intubation was met (Table 1).

**Table 1:** Summary of preoperative airway assessment findings ASA: American Society of Anesthesiologists

Parameter	Finding/value
Mallampati classification	Class II: soft palate, major part of uvula and fauces visible
Thyromental distance	Grossly normal on clinical assessment
Interincisor gap	Clinically adequate mouth opening
Neck mobility	Full: unrestricted active flexion and extension
Body mass index	31.2 kg/m² (Class I obesity)
Predicted difficult airway	No: standard assessment did not identify predictors of difficulty
ASA physical status	II: controlled type 2 diabetes, hypercholesterolemia, mild asthma

On directed questioning, the patient disclosed having received a general anesthetic around five years ago in Somalia for open cholecystectomy and reported that she experienced severe cough and throat pain that lasted for about 12 hours after the general anesthetic. No anesthetic records were available. The history was recorded but at first was not interpreted as being associated with structural airway difficulty, which became evident only during surgery.

Standard monitoring was used. The anesthetic induction drugs included midazolam 2 mg, fentanyl 150 μg, propofol 200 mg, and rocuronium 70 mg. Bag-mask ventilation was used to confirm appropriate mask seal and chest rise; ventilation was achievable with a proper seal around the facemask, though it required relatively high peak airway pressures of 28-32 cmH₂O with tidal volumes of 300-350 mL on the ventilator, and a normal capnograph waveform was present throughout.

Laryngoscopy with a video laryngoscope (McGrath blade, size 3) was performed by the primary anesthetist, which yielded a poor view (Cormack-Lehane Grade III, with only the epiglottis visible). With bimanual external laryngeal manipulation, the view improved to allow visualization of the arytenoids, but the vocal cords remained obscured. This was followed by advancing a gum-elastic bougie; direct visualization of glottic passage was not possible given the limited view. Hold-up was felt but was not relied upon as definitive confirmation of tracheal placement. A 7.5 mm endotracheal tube with a cuff was railroaded over the bougie but encountered subglottic resistance and failed to be advanced. Bag-mask ventilation was immediately restarted, and peripheral capillary oxygen saturation (SpO₂) was maintained between 97% and 99%.

Senior assistance was called in immediately, consistent with DAS 2025 guidance on early escalation within Plan A. A second attempt was made with a tube size 7.0 mm (by the primary anesthetist) over a newly inserted bougie, similarly failing at the subglottic resistance point. This bougie was left in situ following the failed attempt. The senior anesthetist arrived and proceeded with video laryngoscopy, using the previously placed bougie, which remained in tracheal position with hold-up (tracheal placement was not yet definitively confirmed at this stage). A 7.0 mm cuffed endotracheal tube was readvanced over this bougie to the point of subglottic resistance. Then, a deliberate rotational technique was performed: the tube was slowly rotated clockwise, then anticlockwise, while applying gentle concurrent axial pressure to make progressive advancement beyond the subglottic obstruction. Correct positioning was confirmed by continuous waveform capnography and bilateral chest auscultation. The overall time from induction to successful intubation was nine minutes, and SpO_2_ did not drop below 97%. Anesthesia was maintained with sevoflurane in oxygen-in-air, and the incisional hernia repair was carried out, lasting for 74 minutes without any bronchospasm and hemodynamic instability.

Following the completion of surgery, a sugammadex 200 mg (2.5 mg/kg) was injected intravenously. Extubation was performed in the semirecumbent position when the patient was fully awake, obeying instructions and with SpO₂ levels >97% on spontaneous ventilation. She awakened from anesthesia without complications, did not need any oxygen support after 20 minutes, and was moved to the surgical ward in stable condition. Prior to discharge from the hospital, she received both verbal and written difficult airway alerts, documented in the patient record.

## Discussion

This case underscores three important and interrelated points. First, airway symptoms prior to intubation are an underutilized tool for assessing the airway; second, systematic stepwise management can be used when the larynx meets subglottic resistance; third, complete reversal of the neuromuscular block after intubation, plus awake extubation, is important in patients with documented difficult airways.

Sore throat is a common postendotracheal intubation problem. In a study published in 2026, Moulder et al. conducted a systematic review across 43 studies (with more than 21,000 enrolled patients) that found a pooled incidence of postoperative sore throat of 32.4% [6]. Usually, most cases represent transient mucosal irritation caused by cuff pressure or by the movement of the tube; however, symptoms of unusual severity or duration, as in the history of this patient, warrant a more deliberate interpretation. Subglottic contact injury during forced intubation against a structurally narrow trachea would be expected to produce precisely this symptom profile. Similarly, Hibino et al. described an analogous presentation in a 74-year-old female patient in whom asymptomatic subglottic stenosis was discovered only upon encountering subglottic resistance during induction with no history of identifiable warning signs [4]. Together, these reports call for a more inquisitive history of previous anesthetic (with a special focus on symptoms of subglottic difficulty).

The airway management in this case was based on the architecture of the DAS 2025 guidelines [2] that still follow the four-plan (A-D) linear algorithm but with renewed emphasis on maximizing first-attempt success, using capnography at each attempt to confirm successful airway management, and early team activation. The use of video laryngoscopy as the primary laryngoscopic tool is consistent with empirical evidence; a meta-analysis by Araujo et al. confirmed superior first-attempt intubation success with video laryngoscopy over direct laryngoscopy in anatomically challenging patients [7]. The use of video laryngoscopy, in combination with a bougie, is specifically supported by Barnicle et al., who suggest a bougie’s use as a primary adjunct rather than a rescue maneuver in patients, with a 60% improvement in first-attempt success in patients with Cormack-Lehane Grade III or IV airways [8]. In this particular case, bougie placement confirmed the trachea, and tube failure was due to subglottic, not supraglottic, obstruction.

The senior anesthetist's use of the rotational technique is anatomically based. When left oriented, the endotracheal tube is in its natural shape and is at its highest cross-sectional profile at the subglottic area of resistance. Bevels rotate clockwise to anticlockwise, which gradually changes the bevel orientation and allows the tube to be advanced. This technique is described in the literature on bougie-in-channel video laryngoscopy [9] and follows the concept of counterclockwise rotation to prevent arytenoid impingement when railroading. However, rotating the tube does not reduce the diameter of the shaft; it only changes the angle at which the bevel meets the resistance, which may explain why rotation helped the tube pass. Escalation is a logical, least traumatic maneuver to include in the anesthetist's armamentarium and should be considered first on the list when subglottic resistance is confirmed.

Sugammadex use for reversal is due to the lack of proportionality between the risk of residual neuromuscular blockade and its use for reversal in patients with known difficult airways. Postextubation obstruction may occur as a result of incomplete reversal of pharyngeal tone and reduced hypoxic ventilatory drive, especially in those with underlying respiratory disease, for example, asthma [10]. In patients with tracheobronchial stenosis, Lu et al. showed significantly shorter time to extubation and postanesthesia care unit discharge, along with lower incidence and duration of hypotension, with sugammadex vs. neostigmine in a retrospective cohort of patients undergoing rigid bronchoscopy [10].

## Conclusions

This case highlights that a history of prolonged or severe cough and sore throat following a previous general anesthetic should not be dismissed as a routine, self-limiting postintubation symptom. Although no anesthetic records were available in this case, the severity and duration of the reported symptoms raise the possibility that the prior anesthetic may have involved a difficult or traumatic intubation. Such a history may represent an early clinical clue to an evolving difficult airway, and its significance may only become apparent at a subsequent anesthetic, as in this case. We propose that a detailed airway history, specifically enquiring about the nature and duration of postextubation symptoms from previous anesthetics, should form part of routine preoperative assessment, with a low threshold for nasendoscopy or airway ultrasonography where such a history is present. In our patient, definitive endoscopic or radiological evaluation was not pursued postoperatively given the absence of ongoing symptoms and the immediate resolution of the airway difficulty; however, this recommendation is intended to guide future preoperative assessment in similar patients.

In this case, unanticipated subglottic resistance was successfully managed with video laryngoscopy, a reduced-size endotracheal tube, early senior involvement, and a bidirectional rotational technique with gentle axial pressure. This approach should not be generalized as a standard strategy; management of unexplained resistance during intubation must be individualized according to adequacy of oxygenation, quality of laryngeal view, and the suspected level and mechanism of the resistance encountered.

## References

[1] Wang Z, Jin Y, Zheng Y, Chen H, Feng J, Sun J (2024). Evaluation of preoperative difficult airway prediction methods for adult patients without obvious airway abnormalities: a systematic review and meta-analysis. BMC Anesthesiol.

[2] Ahmad I, El-Boghdadly K, Iliff H (2026). Difficult Airway Society 2025 guidelines for management of unanticipated difficult tracheal intubation in adults. Br J Anaesth.

[3] Apfelbaum JL, Hagberg CA, Connis RT (2022). 2022 American Society of Anesthesiologists practice guidelines for management of the difficult airway. Anesthesiology.

[4] Hibino A, Hibino A, Nishimaki H, Denda S (2024). Asymptomatic subglottic stenosis discovered during anesthesia induction and not predicted by preoperative evaluation: a case report. Cureus.

[5] Ellis H, Iliff HA, Lahloub FM, Smith DR, Rees GJ (2021). Unexpected difficult tracheal intubation secondary to subglottic stenosis leading to emergency front-of-neck airway. Anaesth Rep.

[6] Moulder ZJ, Mann J, Bramley P, Heinz J, Wiles MD (2026). Postoperative sore throat: a systematic review. Anaesthesia.

[7] Araújo B, Rivera A, Martins S, Abreu R, Cassa P, Silva M, Gallo de Moraes A (2024). Video versus direct laryngoscopy in critically ill patients: an updated systematic review and meta-analysis of randomized controlled trials. Crit Care.

[8] Barnicle RN, Bracey A, Weingart SD (2025). Managing emergency endotracheal intubation utilizing a bougie. Ann Emerg Med.

[9] See KC, Estaras M, Capistrano R, Wong SH, Sahagun J, Taculod J (2018). Bougie-in-channel intubation technique. Crit Care.

[10] Lu X, Li T, Chen X, Xu M, Wu J, Qiu Y (2023). Sugammadex shortens the time to extubate and discharge from PACU in patients with tracheobronchial stenosis undergoing rigid bronchoscopy procedures: a retrospective cohort study. Front Anesthesiol.

